# The CT-arthrography in the antero-inferior glenoid labral lesion: Pictorial presentation and diagnostic value

**DOI:** 10.4103/0973-6042.39581

**Published:** 2008

**Authors:** Marcello Zappia, Giacomo Negri, Siro Grassi, Cesira Pecoraro, Antonio Rotondo

**Affiliations:** Dipartimento “F. Magrassi - A. Lanzara”, Sezione Scientifica di Diagnostica per Immagini, Seconda Università degli Sudi di Napoli, Italy; 1 Reparto di Ortopedia e Traumatologia, Ospedale Fondazione Betania, Napoli, Italy; 2 Unità Complessa di Radiologia, Ospedale Fondazione Betania, Napoli, Italy

**Keywords:** Arthrography, CT, instability, labral lesion, shoulder

## Abstract

**Objective:**

To present the Computed Tomography (CT)-Arthrography appearance of the most common types of anterior labral lesion and to assess the diagnostic value of this technique in the detection and classification of the antero-inferior labral tears in glenohumeral joint instability.

**Materials and Methods:**

The pre-operative CT-Arthrography records of 43 patients, who underwent surgery for anterior shoulder instability, were retrospectively evaluated independently by two radiologists. The data were compared with arthroscopic results and the diagnostic accuracy of CT-Arthrography was calculated to detect the labral lesion and the agreement between the CT-Arthrography lesions classification and the arthroscopy classification.

**Results:**

The CT-Arthrography sensitivity, specificity and accuracy were: 92% / 89% (reader 1/reader 2), 86% / 86% and 91% / 88% respectively. The CT-Arthrography classification was correct in 86% of cases.

**Conclusions:**

CT-Arthrography appears to be an accurate means for identification and classification of the anterior labral tears and, identifying the labral degeneration, this technique can be very helpful in the selection of patient for arthroscopic stabilization of the shoulder.

The shoulder is the most mobile joint in the human body due to the disproportion between the articular surface of the humeral head and the glenoid; the round surface of the humeral head is about twice the size of the oval and flat glenoid fossa. The stability of the glenohumeral joint is provided by passive and active mechanisms. The active systems are represented by rotator cuff muscles and the tendon of the long head biceps. The passive mechanisms are composed of the capsule-labral structures and ligaments; lesions of one or more of these structures cause pain and instability sensation and are occasionally associated with recurrent dislocation.

Instabilities are more often anterior, occurring in 95% of all patients, in a few cases they are posterior (3%). The remaining 2% of patients have inferior, superior or multidirectional instability.[1] Fractures of the osseous glenoid and humeral head and tears of the labro-ligamentous complex are frequently associated with glenohumeral instability.

The classical labral tear, named “Bankart lesion”, consists of antero-inferior capsule-labral detachments and it is the most common injury associated with instability; usually the labrum is detached from the glenoid rim, the inferior glenohumeral ligament (IGHL) and the capsule are damaged, as is the periosteum of the anterior neck of the scapula.

During recent years, with the development of the arthroscopy procedure, many variants of Bankart lesion have been described in the literature.

Anterior labro-ligamentous periosteal sleeve avulsion (ALPSA) lesion is an avulsion of the antero-inferior glenoid labrum with an intact scapular periosteum; it is characterized by the torn antero-inferior labrum being displaced infero-medially by the inferior glenohumeral ligament.[2] ALPSA differs from Bankart lesion in that the ALPSA lesion has an intact periosteum and the labrum is not detached but it remains attached to the scapula via an intact, but stripped, scapular periosteum.

The Perthes lesion represents a non-displaced avulsed antero-inferior labrum with medial stripping without disruption of the scapular periosteum.[3] In a small percentage of cases, a cartilage lesion of the glenoid surface is associated with labral tear; this lesion is named Glenoid Labrum Articular Disruption (GLAD).[4]

A correct identification of anterior labral tears is necessary for correct treatment and the pre-operative labral lesions categorization can be useful to the orthopedic surgeons.[3,5,6]

Moreover many authors have suggested that, with precise patient selection, the shoulder arthroscopic stabilization can provide high levels of patient satisfaction with low rates of recurrence. The quality and quantity of capsulo-labral tissue and the quantity of glenoid and humeral bone loss are some of the parameters that can change the treatment decision.[7–12]

To correctly diagnose and treat shoulder instability properly, many imaging techniques have been used.[1,13]

The purpose of this study is to present the CT-Arthrography pattern of the different types of anterior labral lesion and to evaluate the accuracy of the technique in the identification and categorization of the anterior glenoid labral lesions using arthroscopy as the reference standard. Many articles have been published in the literature about the accuracy of CT-Arthrography in the detection of labral lesions, but, to our knowledge, none of them have shown images of non-Bankart labral lesion and none of them have evaluated the sensitivity in classifying the anterior labral tears.[14–18]

## MATERIALS AND METHODS

### Patients

We retrospectively examined the CT-Arthrography records of 43 consecutive patients referred for suspected instability of the shoulder. All patients fulfilled the following criteria: 1) CT-Arthrography of the shoulder was performed at our institution (Ospedale Evangelico Villa Betania di Napoli), according to a standardized protocol, between March 2001 and June 2005; 2) all patients underwent surgery or arthroscopy performed by a single specialized **shoulder** surgeon; 3) the indication for surgery or arthroscopy was anterior joint instability; 4) surgery was performed within less than three months of CT-Arthrography; 5) None of the patients had undergone previous shoulder surgery.

Twenty-nine patients were men and 14 were women. Their ages were between 18 and 47 years.

### CT-Arthrography imaging protocol

Arthrography was performed in a radiologic room under fluoroscopic control. Ten to fifteen millilitres of iodinated contrast agent were injected, with a 22G needle, into the shoulder joint, using the anterior approach. To obtain a homogeneous intra-articular diffusion, passive movements were performed, after removal of the needle. CT examination was performed with the arm in a neutral position in all patients; in four cases, additional scans with the arm in internal and external rotation was performed. Informed consent was obtained from the patients before CT-Arthrography. This method was approved by the ethics committee of the hospital.

CT study was performed using GE Pro-speed S Fast scanner (collimation 2 mm; pitch 1); reconstruction at 1 mm was obtained in axial, sagittal and coronal planes. Due to utilization of intra-articular contrast, images were recorded on film utilizing wide window width (2500-3000) and a window level of 300-800.

### CT-Arthrography imaging analysis

CT images were re-analyzed independently by two radiologists (reader 1 and reader 2) expert in musculoskeletal radiology, blinded to patient history, arthroscopic results and the relative proportion of the various surgically findings.

The criteria used to diagnose a labral tear were the following: the identification of contrast material under the anterior labrum and out of the capsule for Bankart lesion; the identification of contrast media under the anterior labrum with an intact capsule for Perthes lesion; the medially dislocation of anterior labrum with an intact capsule for ALPSA lesion; the identification of contrast media within articular cartilage of anterior glenoid surface for GLAD; the absence of labrum or its inhomogeneous morphology and/or density was classified by the radiologists as absent/fragmented labrum.

### Arthroscopy

At arthroscopy the glenoid labrum was normal in 7 patients and abnormal in 36. Of the 36 pathologic labrum, the following types of lesion were found; 16 Bankart lesions; 10 ALPSA; 6 absent/fragmented labrum; 3 Perthes lesion; 1 GLAD of anterior labrum.

### Data analysis

We compared the presence or absence of labral lesion at CT-Arthrography with the surgical findings in each patient and determined the number of true-positive, true-negative, false-positive and false-negative imaging results. The sensitivity, specificity, positive predictive value, negative predictive value and accuracy of the imaging technique in identification labral pathology were calculated.

The CT-Arthrography and arthroscopy classifications were compared evaluating the percentage of agreement and the sensitivities in detection and correctly categorization of the Bankart lesion, ALPSA lesion and absent/fragmented labrum were calculated.

The inter-observer agreement was also evaluated.

## RESULTS

Results are summarized in Tables 1–3.

**Table 1:** Assessment of labral tear on 43 patients*

		Arthroscopy	Total
	(Reference standard)		
	Lesion	Negative	
CT Arthrography			
Lesion	33/32	1/1	34/33
Negative	3/4	6/6	9/10
Total	36	7	43

**Table 2:** CT-Arthrography diagnostic efficacy in detecting labral tear*

Sensitivity	91.6%/88.8%
Specificity	85.7%/85.7%
Positive predictive values	97%/96.9%
Negative predictive values	66.6%/60/%
Accuracy	90.7%/88.3%

* Data are values for reader 1/reader 2

**Table 3:** CT-Arthrography classification and Arthroscopy classifications of anterior labral tear*

Labrum type	Arthroscopy classification (Reference standard)	CT arthrography classification	True positive	True negative	False positive	False negative
Normal	7	9/10	6/6	33/32	3/4	1/1
Bankart	16	15/15	14/14	26/26	1/1	2/2
ALPSA	10	10/10	10/10	33/33	0/0	0/0
Perthes	3	2/1	1/1	39/40	1/0	2/2
GLAD	1	1/1	1/1	42/42	0/0	0/0
Absent/Fragmented	6	6/6	5/5	36	1/1	1/1

* Data are number of labral tear findings for reader 1/reader 2

Considering arthroscopy as the reference-standard, the CT-Arthrography gave a high accuracy value of 90.7% / 88.3% (reader 1 / reader 2), with a sensitivity of 91.6% / 88.8% and specificity of 85.7% / 85.7% in detecting anterior labral lesions.

**Figure 1:**
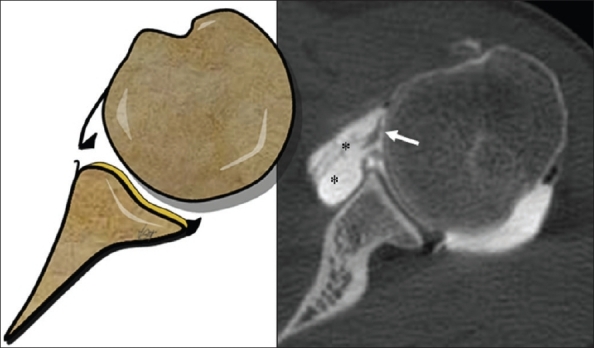
An axial CT-Arthrography image of a 32-year-old man, demonstrates the capsular lesion (*asterisks*) with the detached anterior labrum (*arrow*), which constitutes the fibrous Bankart lesion

**Figure 2:**
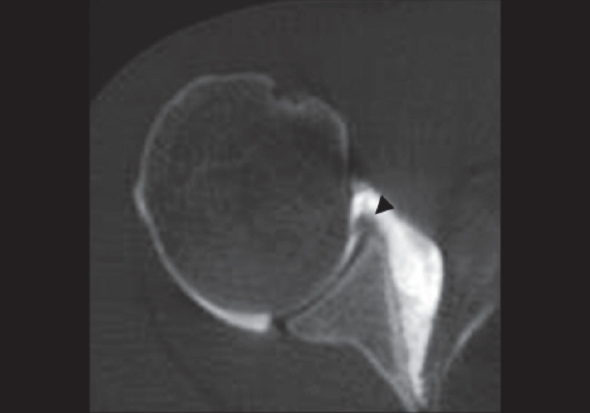
Axial CT-Arthrography image of the 27-yearold man with arthroscopy proved Bankart lesion. The CT-Arthrography image shows only the capsular tear, but no contrast media between the labrum and the glenoid rim is present (*arrow*). It was interpreted as normal labrum

Of 16 Bankart lesions identified at arthroscopy; 14 / 14 were correctly diagnosed by CT-Arthrography [Figure 1]; 1 / 1 lesion was identified but it was categorized as absent/fragmented labrum; in one case the Bankart lesion was not identified by both radiologists [Figure 2]; one false-positive for Bankart lesion was found by reader 2.

The sensitivity in identification of the Bankart lesion was of 93.7% and the sensitivity in categorization was of 87.5% for both radiologist.

**Figure 3:**
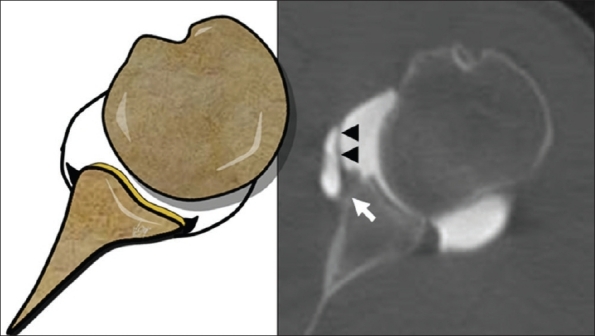
Axial CT-Arthrography image of a 25-year-old man with arthroscopy proved ALPSA lesion. The anterior labrum is displaced medially (*arrow*) and the capsule is intact (*arrowed*)

**Figure 4:**
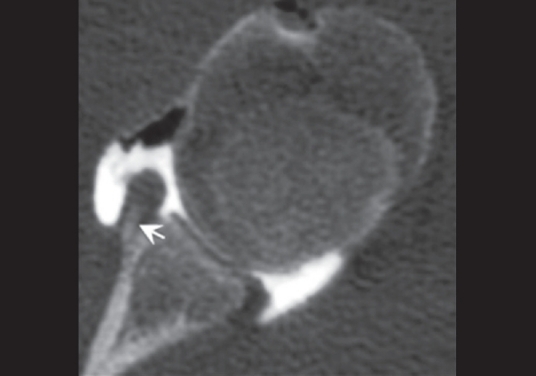
Axial CT-Arthrography image of a 30-year-old man with arthroscopy proved ALPSA lesion and demonstrates the periosteal reaction (*arrow*) due to periosteum stripping

Ten ALPSA lesions were identified at arthroscopy and all of them were correctly identified and classified by CT-Arthrography with a sensitivity value of 100% [Figures 3,4].

**Figure 5:**
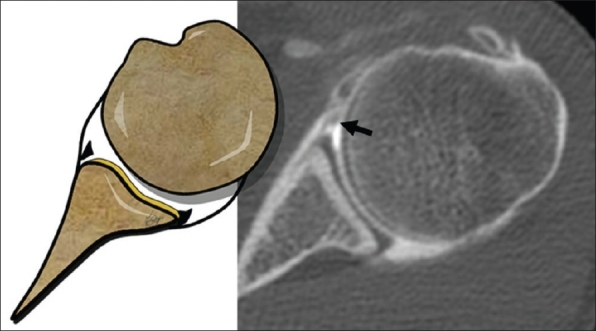
Axial CT-Arthrography image of a 26-year-old man shows non-displaced Bankart lesion, otherwise known as Perthes lesion (*arrow*)

Of three Perthes lesions diagnosed by arthroscopy, only one was identified at CT-Arthrography; the lesion was clearly diagnosed by the images of the scans with the arm in external rotation [Figure 5]; in both cases in which the lesion was not identified, additional scans with the arm in internal and external rotation were not performed; one false-positive for Perthes lesion was found by reader 1.

**Figure 6:**
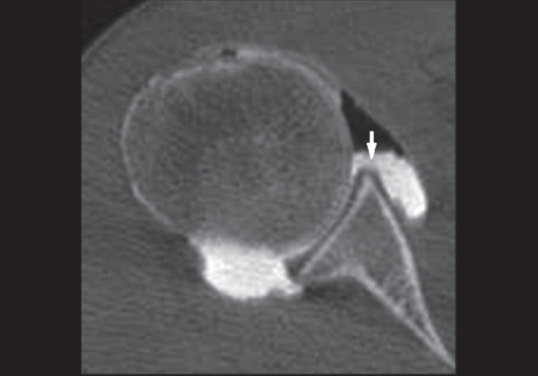
Axial CT-Arthrography image of a 33-year-old man with arthroscopy proved absence of the antero-inferior labrum (*arrow*)

In six cases the surgeon described the labrum as degenerated; in five of these cases the radiologists diagnosed the tears and correctly classified them as fragmented/absent labrum [Figure 6]; in the remaining case radiologist 1 identified the lesion but categorised it as Bankart lesion and reader 2 classified the labrum as normal diagnosing only the capsular tear. The sensitivity in identification and classification of the absent/fragmented labrum was 83.3% / 100% and 83.3% / 83.3% respectively.

**Figure 7:**
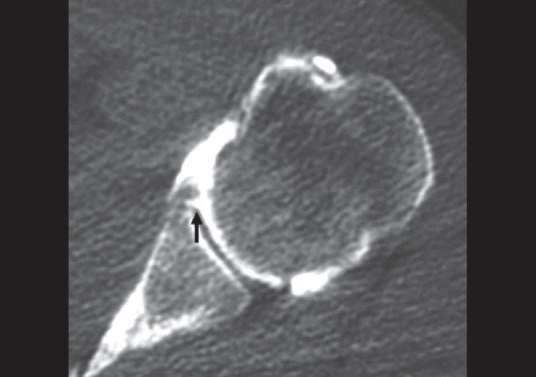
Axial CT-Arthrography image of a 40-year-old man wit arthroscopy proved GLAD lesion. Note the presence of contrast media in the glenoid cartilage (*arrow*)

The one GLAD lesion [Figure 7] was correctly identified and classified.

The arthroscopic and CT-Arthrography classifications were in agreement in 86% of cases (37/43 cases).

Inter-observer agreement for classification of labral lesions was 95.3%.

## DISCUSSION

The difference in size between the glenoid fossa and humeral head allows a wide range of motion in the shoulder joint. The peri-articular components help provide stability of this unstable structure.

In 1938, Bankart considered the labrum an essential component in the prevention of shoulder dislocation;[19] Turkel *et al*, in 1981, investigating the mechanism preventing joint dislocation, found that the inferior glenohumeral ligament labral complex is the primary anterior stabilizer of the shoulder when the arm is at 90° of abduction and external rotation.[20] Successively, several authors have demonstrated that lesions of the glenoid labrum and its capsular attachment, can be associated with and cause shoulder instability and symptomatology.[21]

To correctly diagnose and properly treat shoulder instability, many imaging techniques have been used such as routine radiography, ultrasound, Computed Tomography (CT), CT-Arthrography, conventional Magnetic Resonance (MR) and MR-Arthrography.[13,22–24]

Many authors indicate MR-Arthrography as superior to other imaging methods in evaluation of the glenohumeral joint, but the use of this technique is limited by several contraindications, such as claustrophobia, metallic objects inside the body or pace-makers.[1,25] Moreover, in our clinical practices, CT-Arthrography is required more than MR-Arthrography, probably because of the higher diagnostic confidence of the orthopedic surgeons with CT images, the higher distribution of CT scanners in our region and the possibility of this method to evaluate the exact bone glenoid loss.[26]

Several studies have described the diagnostic accuracy of the imaging methods in identification of labral lesion but, to our knowledge, only Waldt *et al*, have been calculated the accuracy in the categorization of the labral lesions at MR-Arthrography.[27] Furthermore, no studies on CT-Arthrography anterior labral tear classification have been published.[14–18,25]

In this study the CT-Arthrography has demonstrated high values of sensitivity (91.6% and 88.8% respectively for each reader) in detection of anterior labral tears.

Previous CT-Arthrography studies show a large discordance of results, with a sensitivity varying from 73% to 92%.[14–18] Therefore, it is difficult to make a proper comparison with our results.

Instead, our results are comparable with those of previous investigations on the diagnostic accuracy of MR arthrography.[1,27]

To correctly diagnose Bankart lesion, the CT-Arthrography technique has shown a high sensitivity, identifying and categorizing the lesions in 93% and 87% of cases respectively; the contrast agent creates a high density line between the glenoid rim and its detached labrum, showing the lesion clearly [Figure 1].

In the older Bankart lesion, false negatives could be found; the presence of fibrous scar can prevent the contrast media passage and it may mimic an intact labrum.

Correct identification and classification of ALPSA lesion was established, in 100% of cases, with a sensitivity value higher than MR-Arthrography.[27]

The intact capsule, allows a good contrast media concentration inside the joint, showing the medially dislocated labrum. Moreover, periosteal reaction of the anterior aspect of the glenoid, caused by periosteum stripping, could be considered a specific indirect sign of ALPSA lesion [Figure 3].

CT-Arthrography has shown a very low sensitivity in detection and characterization of the Perthes lesion. The intact capsule and the undisplaced labrum create difficulties in recognizing this type of lesion. Many authors have already suggested that scans with the arm in external rotation or in external abduction rotation (ABER) position are necessary to show the displacement of the labrum.[27,28] In our retrospective study, only a few patients were studied with additional scans, therefore no statistical results can be made.

In our study, in six cases the surgeon described the anterior labrum-ligamentous complex as degenerated, with a poor quality and quantity of tissue; therefore the arthroscopic stabilization procedure could not be performed. CT-Arthrography has shown a sensitivity of 83% / 100% in identification of this abnormal condition and 83%, for both readers, in categorizing it as fragmented/absent labrum.

To our knowledge, the distinction of Bankart lesion from ALPSA, Perthes or GLAD may be useful but not necessary for a treatment decision, but, many several authors have suggested that a degeneration or absence of the antero-inferior capsulo-labral complex is an important criteria for treatment decision.[7–12]

In this study the authors have demonstrated the high sensitivity value in diagnosing the labrum degenerative change; other authors have demonstrated the possibility of the CT to quantify the exact glenoid bone loss;[26] the authors suggest that the use of CT-Arthrography as a pre-operative imaging technique could be very helpful in treatment planning.

Here we mention some limitations in our study. First, although arthroscopy was the best reference standard achievable in this study setting, it is an operator-dependent technique and some labral lesions are difficult to see at this type of procedure.[28]

The second limitation was the small number of cases with intact labrum and the absence of control subjects; the specificity value in identification of labral tears might not be correct.

Another limitation is the small number of cases of Perthes and GLAD lesions; again the sensitivity in detection of Perthes lesion must be studied with scans in ABER position and in our retrospective evaluations it was not possible. Therefore no significant statistical conclusions about diagnostic sensitivity and inter-observer agreement for identification of these types of lesions, can be made.

The fourth point is that the patients with acute and chronic instability were not studied as different groups.

In conclusion, CT-Arthrography is a valid technique to identify and classify anterior labral lesion occurring in anterior shoulder instability. As it can detect the degenerative phenomena of the labrum and a quantify the glenoid bone loss, CT-Arthrography can be very helpful in the selection of patients for arthroscopic shoulder stabilization procedure.
